# Immediate function on the day of surgery compared with a delayed implant loading process in the mandible: a randomized clinical trial over 5 years

**DOI:** 10.1111/clr.12279

**Published:** 2013-10-23

**Authors:** Asbjorn Jokstad, Hassan Alkumru

**Affiliations:** Faculty of Dentistry, University of TorontoToronto, ON, Canada; Department of Clinical Dentistry, Faculty of Health Sciences, UiT The Arctic University of NorwayTromsø, Norway

**Keywords:** alveolar bone loss, cantilever units, dental implants, fixed prosthesis, marginal bone level, radiographic evaluation, randomized clinical trial

## Abstract

**Objectives:**

To appraise the feasibility of loading four implants with a pre-existing denture converted to a fixed dental prosthesis (FDP) on the day of implant surgery compared with waiting for 3- to 4-month healing.

**Methods:**

Patients with an edentulous, fully healed mandible were recruited in a faculty clinic to partake in a blinded two-arm parallel randomized controlled trial (RCT). The participants received four parallel intraforamina mandibular implants with a moderately rough titanium surface (Brånemark System Mk III or Mk IV TiUnite; Nobel Biocare AB, Göteborg, Sweden). The implants were loaded on the same day by converting the participants' pre-existing denture in the experimental group. The implants were placed using a one-stage surgery procedure, and the participants' pre-existing denture were soft-relined in the control group. For both groups, the permanent 10- to 12-unit FDP consisting of a type-3 cast precious alloy veneered with acrylic and artificial teeth was placed 3–4 months after implant surgery. All participants have been recalled annually for 5 years for appraisal of bone loss and registration of adverse events.

**Results:**

Thirty-five of the original 42 participants (83%) returned for clinical and radiological examinations at the 5-year follow-up recall. No selective dropout or specific reasons for dropout was identified in the two study arms; leaving *n* = 17 (*Intention-to-treat group*, ITT) in the experimental group, alternatively *n* = 13 as *per protocol group* (PP), and *n* = 18 participants in the control group (ITT = PP). At study commencement, five of the participants assigned to the experimental group did not receive their planned intervention. In the control group, one implant failed to osseointegrate and another failed due to bone loss after 5 years. The crestal bone level changes over 5 years were identical in the experimental and control groups, that is, 1.2 mm (SD = 0.7). There were no differences between the two study arms with regard to incidence of biological and technical adverse events.

**Conclusions:**

Implants in the anterior mandible loaded immediately with a converted pre-existing denture appear to yield analogous clinical outcomes compared with waiting for 3–4 months over the first 5 years following implant surgery.

Titanium implants placed in the jaw to retain a dental prosthesis were previously left to heal subgingivally without functional loading for a minimum number of months depending on the bone quality. Some investigators and clinicians still follow this procedure, originally established by the Brånemark research team in the 1970s (Brånemark et al. 1977). The precautionary approach is based on the concern that subjecting a newly placed implant to functional loading will compromise the osseous integration of the implant. Intensive research on bone healing – physiology and remodeling processes within basic and clinical sciences – has gradually provided a better understanding of the osseointegration of dental implants (Jokstad 2009). The better understanding of osseointegration has led to the one-stage surgeries and to minimize the period between the implant surgery and the prosthesis placement (Szmukler-Moncler et al. 2000).

A perceived advantage of immediate loading following implant placement is that the patient will be able to quickly resume oral functions and appearance and also to avoid a second surgery to expose the implant. Several clinical studies suggest that immediate or early loading of implants with a fixed prosthesis is feasible. However, estimates of relative risks or odds ratios of adverse outcomes compared with a conventional approach are uncertain because only a distinct minority of studies have been designed as a randomized clinical trial (RCT) with adequate study power and quality of reporting, for example, according to the CONSORT criteria (Esposito et al. 2003, 2004, 2007, 2009). Moreover, such estimates are confounded further by the range of RCT study design variations regarding, for example, number of implants to support the supra-construction, the intraoral location of the implants, the interimplant distances and angulations, the implant dimensions with regard to diameters and lengths as well as the actual implant brand and surface roughness (Jokstad & Carr 2007). In summary, we can advise a patient prior to obtaining informed consent that early and immediate loading of implants may be successful, but the relative risks of adverse outcomes compared with using a delayed loading approach remain uncertain. Such data can only reliably obtained by carefully planned and properly conducted RCTs.

In 2000, Nobel Biocare AB (Göteborg, Sweden) developed a new moderately rough titanium surface named TiUnite by the use of spark anodization. The initial data from the i*n vitro* and animal studies, as well as several short-term clinical data (Glauser et al. 2003, 2005; Rocci et al. 2003; Attard et al. 2005; Jungner et al. 2005; Watzak et al. 2006) suggested that the implants with the TiUnite surface outperformed the identical implants with a turned surface made by this implant manufacturer. Given the existing literature in 2006, it was considered scientifically and ethically acceptable to initiate a trial to examine the feasibility of adopting an immediate loading protocol for implants with a TiUnite surface in the anterior mandible.

Various approaches have been used to fabricate a full-arch fixed prosthesis to immediately load implants. Some practices have been to take an immediate impression for stone casts and acrylic bridgework with or without metal reinforcement, or alternatively have acrylic bridgework with or without metal reinforcement made in the laboratory before placing the implants (Maló et al. 2003; Jokstad & Carr 2007). A third approach is to transform the patient's pre-existing conventional denture into a provisional fixed prosthesis, which several investigators have used with apparent good clinical success (Calvo et al. 2000; Cooper et al. 2002; Chee & Jivraj 2003; Aalam et al. 2005). The various fabrication procedures have inherent benefits and disadvantages regarding costs, logistics and sources of errors. However, a perceived benefit of the latter approach is that the occlusion is well established and familiar to the patient; moreover, the clinicians and the patient have the time to decide on the optimal tooth shade and dimensions, besides the ability to establish the correct vertical dimension of occlusion, occlusal table width and vertical and horizontal maxilla–mandibular occlusal relationships.

Hence, a clinical study was designed with the objective to appraise the clinical outcomes following loading of four implants in the area between the two mental foramina of the mandible with a full-arch fixed dental prosthesis (FDP) converted from the patient's pre-existing denture on the same day as the implant placement. The approach used for the control patients was to wait for 3–4 months to allow healing after the implant placement surgery. The study hypothesis was that there would be no differences in clinical performance over 5 years between the FDPs and implants when these were loaded immediately versus loading subsequent to 3 months of healing.

## Materials and methods

The study protocol and patient information documentation were approved by the University of Toronto Research Ethics Board (#22797) in 2006. The case report forms including adverse event forms were developed in accordance with ISO 14155 guidelines. All clinical procedures were provided by a certified oral–maxillofacial surgeon (OMS) and one or more prosthodontists. An independent researcher was responsible for the study participant randomization and allocation concealment.

### Power calculation and study arm sample size considerations

Sample sizes were calculated for a two-sided test to compare two independent groups using the formula: 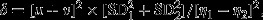 with [η_1_ − η_2_] being the difference between the means for two representative study samples and SDs their standard deviations. Data on immediately loaded implants in the mandible were identified in a study that presented bone loss on sixty implants placed in 20 patients (De Bruyn et al. 2001). The mean of bone loss during the first year was 0.9 mm (SD = 1.1). Data of comparable implants with a delayed loading protocol were identified in a paper published by Engquist et al. (2004), where the bone loss was based on 113 implants placed in 30 patients, that is, 1.73 mm (SD = 0.6). With a μ = 0.05 and 80% study power and based on these data, the sample size was estimated to be 2 × 18 participants. In anticipation of participant dropout, the proposed number of 2 × 21 participants was selected.

### Participant population

**Figure 1:**
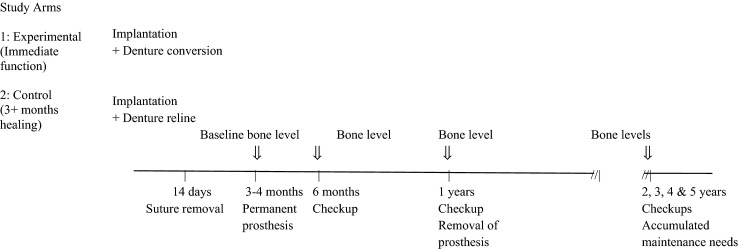
Progress plan of the trial over the 5 years.

All study participants were recruited from the pool of patients referred for advanced prosthodontic treatment from general practitioners to a university faculty prosthodontic graduate clinic. Patients with an edentulous mandible healed more than >3 months after the tooth extraction with a bone ridge width ≥7 mm and a bone height ≥8 mm and who desired a FDP were eligible for study participation. An augmentation procedure was allowed, but would require at least 6 months healing prior to implant surgery. Potential participants were informed by graduate clinic faculty and support staff of the overall requirements and procedures of the clinical study, the nature of the planned treatment, alternative procedures and the potential risks, possible complications and benefits of the proposed treatment. They were also advised of the need for the prescribed follow-up visits for their ongoing care and for the collection of relevant study data as shown in Fig.1 and that they could withdraw from the clinical study at any time without any consequences. All patients had read, understood and signed the written informed consent form at least 7 days before implant surgery. Once written consent was obtained, a clinic staff prosthodontist verified that the participant satisfied several inclusion and exclusion criteria for participation (Table1) before enrolling the patient into the study. Additional exclusion criteria applied by the OMS surgeon during the implant surgery were insufficient bone or inability to place four implants between the two mental foramina. In these instances, the participant was withdrawn from the study.

**Table 1:** Inclusion and exclusion criteria used in the current trial

Inclusion criteria	Exclusion criteria
Patient at least 18 years or older Edentulous mandible at least 3 months before date of implant surgery Eventual previous GBR/GTR procedures carried out at least 6 months prior to implant surgery Adequate oral hygiene Good dexterity for oral hygiene maintenance Absence of local inflammation, mucosal disease Adequate bone quality and quantity for placement of four implants between the two mental foramina without need for bone augmentation simultaneous with implant placement Commitment to follow-up examinations	*Systemic* Conditions requiring prolonged steroid use Presence of conditions requiring prophylactic use of antibiotics History of leukocyte dysfunction or deficiency, bleeding disorders, neoplastic disease, renal failure, uncontrolled endocrine disorders Use of any investigational drug or device within 30-day period immediately prior to implant surgery History of radiation therapy History of alcohol or drug abuse Patient infected with HIV Smoking >10 cigarettes per day or tobacco equivalent Visible indications of severe bruxism Advanced age and/or compromised general health so the surgical appointments are too demanding
	*Local* Need for site augmentation through grafting

### Pre-implant treatment procedures

Pre-implant treatment procedures included clinical examinations, appropriate medical history, determination of concomitant medication usage and appropriate radiographs such as orthopantomogram or cone-beam CT. Any remaining teeth and roots were extracted at least 3 months prior to the scheduled implant surgery. In case of eventual previous bone grafting, the scheduled implant surgery had to be conducted after at least 6 months. Any existing removable full dental prostheses were critically evaluated by both the participant and the clinician and, if needed, optimized for esthetics, fit and occlusion if possible. If deemed irremediable, standard fabrication routines were used to fabricate a new mandibular and/or maxillary removable prosthesis using heat-cured acrylic resin (Biolon; Dentsply Prosthetics, York, PA, USA) combined with prefabricated teeth made of acrylic resin (Biodent; Dentsply Prosthetics). The occlusion was adjusted to be balanced in latero- and protrusion, and continuous adjustments were made until complete participant satisfaction with the denture(s) fit and esthetics.

### Surgical procedures

Each participant received four vertically parallel implants placed with a symmetrical spread to support a 10- to 12-unit FDP. All implants were placed between the two mental foramina with the most distally placed implants in the 34/35 and 44/45 regions.

The implant surgeries were performed by a single OMS under sterile conditions in an outpatient environment. Prophylactic antibiotic therapy was given at the surgeon's discretion according to clinical routine considerations. Implant surgery was performed under local anesthesia (2% lidocaine, 1 : 100,000 epi, sometimes in combination with 4% Articaine, 1 : 200,000 epi). The surgeon reflected full-thickness mucoperiosteal flaps. Surgical retractors were not typically used to avoid unnecessary soft-tissue damage. Careful ridge alveoloplasty was performed in a few cases to achieve a flat bone surface of sufficient width. In situations with a narrow ridge crest, reduction was made to obtain the necessary width of at least 7 mm in the buccal–lingual direction.

All implants were Brånemark System Mk III or Mk IV implants with a TiUnite surface (Nobel Biocare AB) having diameters of 3.75 or 4.0 mm and lengths of 10, 11.5, 13 and 15 mm. The Mk IV implants were used (*n* = 22) instead of Mk III implants (*n* = 146) in situations when the surgeon opted for better primary stability in predominantly loose trabecular bone.

The surgeon followed the implant manufacturer's guidelines for standard procedures relating to bone drilling sequence, site preparation and placement of the implants. Drilling procedures were performed with light hand pressure and sink depth controlled with a depth gauge. Screw tapping or countersinking conditional on the bone quality was left to the discretion of the surgeon. The recipient site was flushed with sterile saline, and the implant placed using an insertion device. The manufacturer recommendation that immediate loading should be feasible if the insertion torque lies within a range 35–45 N cm was not strictly followed. The implant insertion torque was not measured, and the initial stability of the implants was assessed using a manual torque wrench set to 20 N cm. Based on the hand torque testing, the surgeon reported the implant stability as acceptable or inadequate. If the primary stability of any of the four implants did not reach 20 N cm, the participant was *per protocol* (PP) to be reallocated into the control arm of the study, that is, with a 3- to 4-month unloaded healing period.

Periapical radiographs were taken routinely immediately after implant surgery for implant location verification purposes. The randomization envelope seal was first broken once the surgical implants had been placed.

### Randomization

Participants were allocated to either the experimental or control group following a randomization list that had been generated by an independent researcher. No *pre hoc* blocking or stratification rules were applied in the study. Each participant was assigned a unique participant number, and the allocation code was kept in numbered sealed opaque envelope originating from the independent researcher. After opening, following the implant placement, the opaque envelope was kept as a source document for later verification against the randomization list. Any discrepancies between the list and source document records would have led to case exclusion, but no such events were discovered when a control was made once the participant accrual period had ended.

### Immediate post-implant surgery

**Figure 2:**
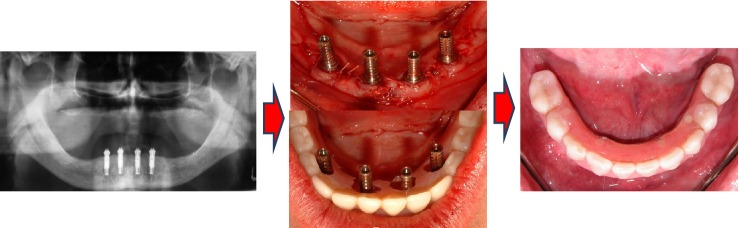
Illustrative details of the conversion from a pre-existing conventional denture to a 10-unit fixed dental prosthesis in the experimental study arm.

The implant site was sutured, and the participants were instructed not to brush in the treated area, and to rinse twice daily for one minute with chlorhexidine digluconate for plaque control. NSAID analgesics, predominantly acetaminophen or ibuprofen, were given as required for pain control. According to study arm allocation, two different prosthodontic protocols were followed. In the experimental group, the removable prosthesis was converted into an implant-supported FDP. Multiunit abutments (Nobel Biocare AB) with appropriate heights were first secured on the implants and fitted with titanium temporary cylinders. A rubber dam was perforated and adapted around the implants and abutments to protect the mucosa. Access openings on the removable prosthesis were identified using a silicone elastomeric material (Fit Checker; GC America, Alsip, IL, USA). Following the grinding of holes, the temporary cylinders were secured to the denture acrylic prosthesis using cold-cure acrylic (Jet acrylic; Lang Dental Manufacturing Co., Inc, Wheeling, IL, USA). The prosthesis was then sent to the laboratory to complete the cylinder–prosthesis combination, polymerization and polishing. The prostheses were also relieved of the distal ends bilaterally to allow for maximum 12-mm-long cantilevers, as measured from the distal abutment center (Fig.2). The prosthesis was reinserted on the same day as the implant surgery and secured with the 4 bridge screws (TorqTite; Nobel Biocare AB) torqued to 15 N cm. Finally, the occlusion was adjusted to anterior and cuspid guidance using 6 μm shim stock between the teeth. Post-operative instructions for intraoral cleaning were given to the participant.

In the control group, the implants were fitted with healing abutments extending flush with the mucosa. The existing removable prosthesis was relined using a soft-reline plasticized acrylic-based material (Coe-Soft; GC America) ensuring no impingement of the healing abutments.

In cases where a removable prosthesis was opposite the study implants, the participant was advised to not wear the opposing prosthesis for 2 weeks after surgery. After approximately 10 days, the participants were recalled for suture removal. All participants received instructions on appropriate home care.

All participant complaints or any complications resulting from a change in health status or any implant-related complications such as pain, paresthesia or peri-implant infection were recorded and monitored from the implant surgery date.

### Permanent prosthodontic procedures

The permanent FDP was completed within 3–4 months after the implant placement using routine restorative treatment procedures (Zarb & Jansson 1985). In brief, standard pickup impression copings were fitted onto the multiunit abutments and removed adhering to a polyether impression material (Impregum; 3M ESPE, Seefeld, Germany) using an open-tray impression technique. Dental stone casts were made with implant replicas, and the accuracy of the replica placements was verified intraorally with the use of an intraoral acrylic jig (Duralay; Reliance Dental Mfg. Co, Worth, IL, USA). The participants’ maxilla–mandibular vertical relation was established using wax rims. Centric relation recordings were used for mounting the stone casts on an articulator. Artificial tooth setup was established with anterior and cuspid guidance in medio-laterotrusion and freedom in centric. The default occlusal setup included 10 teeth, albeit in a few situations due to the maxillary occlusion 11 or even 12 teeth were placed to maintain a balanced occlusion. The wax-up dentitions were tried intraorally and following participant approval indexed for final processing.

### Dental laboratory procedures

All technical work was made by one dental technician employed in the faculty in-house dental laboratory using the manufacturer's standard prosthetic components and in accordance with the manufacturer's instructions. In brief, burnout copings (Gold Coping Multi-Unit; Nobel Biocare AB) were adapted over the implant replicas in the dental stone cast (Fujirock; GC America) and bound with wax sticks. Castable retention pins and beads were incorporated in relevant locations of the wax framework to support the acrylic teeth and veneering. The extension of all cantilevers was a maximum of two tooth units or 15 mm.

A passive fit test of the waxed frameworks to the master cast was performed by manually tightening a screw at the most distal end. Inaccuracies were adjusted by cutting and melting until a passive fit was obtained. The wax models were sprued and invested using a carbon-free phosphate-bonded investment compatible with the precious gold alloy used for casting the prosthesis framework (Olympia; Jelenko, Armonk, NY, USA; Au: 51.5%, Pd: 38.4%, In: 8.5%). The framework was cast in one piece and ground to desired shape. The invested sprue was heated to 950°C for 120 min before being transferred to a casting machine (Multicast; Degussa, Pforzheim, Germany). After casting and cooling the framework, it was divested and sandblasted with 50 μm aluminum oxide powder avoiding damage to the implant cylinder platform areas. Sprue formers and casting nodules were removed under magnification.

Passive fit of the metal frameworks was measured by manual tightening, that is, about 10 N cm, of one screw at the terminal abutment on the master model and examined in an optical stereo-microscope at ×15 magnification. Non-passive fit revealed as gaps were corrected by sectioning the cast and soldering using a solder for the specific alloy (Olympia Pre; Jelenko).

**Figure 3:**
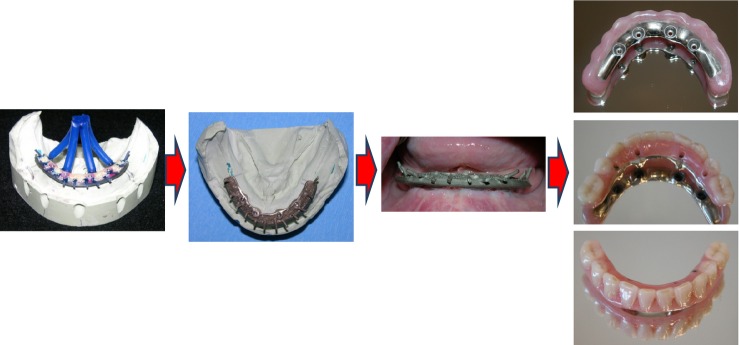
Illustrative details of the fabrication of the permanent full-arch metal-reinforced acrylic fixed dental prosthesis.

The veneering of the metal substructure was heat-cured acrylic resin (Biolon; Dentsply Prosthetics) processed onto the frameworks following standard laboratory procedures combined with prefabricated teeth made of acrylic resin (Biodent; Dentsply Prosthetics; Fig.3).

### Delivery of the permanent FDP

Before loading the implants with the permanent FDP, each implant was checked for mobility by direct finger manipulation around the implants and by evaluating the tapping sound made with a hand instrument.

The FDP was attached to the abutments using standard gold prosthetic screws using the 15 N cm screw torque per the manufacturer's recommendation. The screw holes were filled with a cotton pellet or plastic tape topped with a light-curable composite resin (Tetric; Ivoclar Vivadent, Schaan, Liechtenstein), adjusted and polished. The occlusion was readjusted with anterior and cuspid guidance in medio-laterotrusion and freedom in centric using shim stock and/or articulator paper.

All participants were encouraged to maintain good oral hygiene and instructed in the use of individually suited interproximal brushes (TePe, Malmö, Sweden).

Following the placement of the FDP, radiographic film holders (Rinn XCP; Dentsply Rinn, Elgin, IL, USA) were customized to ensure a position of the film tangential to the indicator arm by adapting the film holders to the occlusal surface using a heavy body elastomer (Express; 3M ESPE). Radiographs were taken with the film placed parallel to the implants, and the x-ray beam directed perpendicular to the implants to include at least two coronal implant threads. The film holders were marked and kept for future recordings, enabling subsequent repeat standardized periapical radiographs from the time of permanent loading.

### Primary and secondary outcomes

The primary outcome of interest in the clinical study was the amount of crestal bone level changes occurring between the time of loading the implants with the final prosthesis (baseline) and different time points, as measured on the standardized periapical radiographs. Secondary outcomes of interest were the incidence of any types of technical and biological adverse events, including implant mobility, peri-implant radiolucency, peri-implant recurrent infection, structural failure of the implant and framework adverse events.

### Clinical and radiological follow-up examinations

Participants were recalled for examinations 6 months after prosthesis delivery and annually up to 5 years from baseline. Standard clinical extra- and intraoral examinations were performed by a trained prosthodontist, including assessment of periodontal health. Blinding of the clinical examiner to the digital participant chart information was not feasible. At the sessions before and including 1 year, the FDP was removed and each implant was checked individually for peri-implant status. At the subsequent recalls, implant mobility was measured indirectly by movement of the FDP combined with peri-implant radiographic findings and/or clinical signs and symptoms.

Oral hygiene was assessed using sulcus bleeding, plaque index and oral hygiene per established criteria (Mombelli et al. 1987). If indicated, the FDP was cleaned of plaque or calculus, and the importance of maintaining an adequate oral hygiene level was reinforced.

### Radiographic measurements

**Figure 4:**
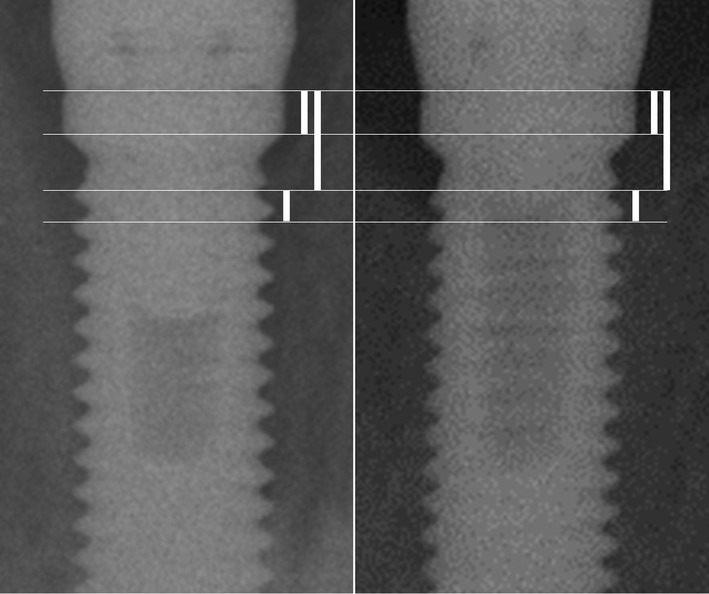
Details of radiographic bone measurement points using a Brånemark System Implant with a regular platform (ø = 3.75 mm). Vertical bars from top of shoulder to horizontal part of the shoulder = 0.8 mm; First thread = 1.8 mm; Second thread = 2.4 mm; Third thread = 3.0 mm etc. The interthread distances = 0.6 mm. Other dimensions apply for the narrow and wide platform implants.

Standardized periapical radiographs using the customized film holders were taken at baseline and at all follow-up examinations (Fig.4). An attempt was also made to evaluate potential crestal bone changes around the implants on the non-standardized periapical radiographs taken on a routine basis for implant placement verification purposes immediately following the implant surgery, and the baseline radiographs taken 3 months after the implant surgery. The same type of film was used throughout the clinical study for consistency. Individual radiographs were digitized using a HP Scanjet 8300 Professional Image Scanner (Hewlett-Packard, Palo Alto, CA, USA). A public domain image processing software (ImageJ; US National Institutes of Health, Bethesda, MD, USA) was used.

Bone level measurements were made blinded by an independent assessor (H.A.) who was unaware of the study arm participant status. The blinded assessor was calibrated with the lead author, and repeat measures were carried out until the intraclass correlation was >95% prior to commencing the measurements. Mean values were used and repeated if the two measurements deviated more than 0.5 mm. Vertical distances in millimeters from the implant shoulder to the most apical initial point of first visible bone contact were measured for both proximal sites using the measurement tool function of the ImageJ software. Eventual misalignments of the film planes relative to the implant long axis were accounted for by calibrating the software for each measurement to some dimensions of the known implant, reliably available in the manufacturer's product catalog (Nobel Biocare 2012). Sporadic repeat measurements were conducted on a random basis to verify that there was no measurement drift during the 5-year study period.

### Statistical analyses

Purely descriptive statistics were used to depict characteristics of the study sample, as well as measured outcomes using either the participant or the implant as the statistical unit. Parametric analyses and nonparametric when appropriate were used to test for statistical differences regarding (I) radiographic bone loss from date of loading with the final prosthesis and (II) type and time to event of any biological and technical complication, applying this first on the implants as the statistical unit and next on the participant level. When the participant was considered as the statistical unit, a complication with any of the four implants or the suprastructure was recorded as an event. All statistical analyses were carried out using SPSS statistical software version 18 (SPSS Inc., Chicago, IL, USA).

## Results

In total, 51 individuals responded to the invitation for participation. Following the provided information about the study protocol and participation requirements, 45 consented to participate. At the time of the initial clinical screening, it was discovered that three consenting participants did not have adequate bone amount. All three participants declined to undergo bone augmentation procedures and wait at least 6 months before implant surgery. Hence, 42 participants without any needs for bone augmentation were randomized into the two study arms of the trial.

**Figure 5:**
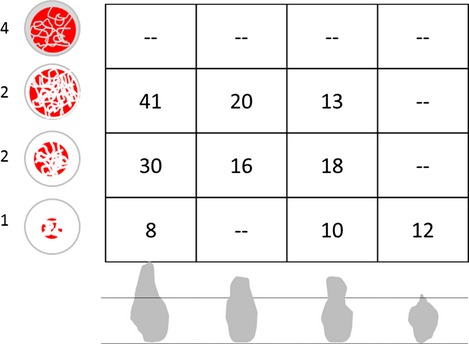
Implants placed in the trial according to bone quality (1–4) and mandible shapes (Tapered – Parallel – Undercut – Knife-edge; *n* = 168 implants).

The participants in this trial received their implants in the period between March 2006 and April 2007. These were invariably placed symmetrically in the mandible in the central/lateral as well as in the first/second premolar areas into bone quality and mandible shapes as shown in Fig.5.

At the time of the implant surgery, it was discovered that one participant allocated to the experimental group had inadequate space to place four implants between the two mental foramina. A second participant in the experimental study arm declined following the surgery to follow the immediate loading protocol, despite initial consent, and was therefore reallocated to the control group. In a third participant, the surgeon failed to obtain adequate minimum primary stability (<20 N cm) of one of the implants. This participant continued using his/her soft-relined denture and waited 4 months before proceeding with loading the implants with a permanent FDP.

Over the course of the post-operative healing period, three participants in the experimental group experienced each that one implant failed to osseointegrate. One of these participants declined further care. The two others underwent a second surgery to receive one new implant each. They continued to use their already converted denture on the remaining three implants, while the new implant was left to heal for 3 months.

**Figure 6:**
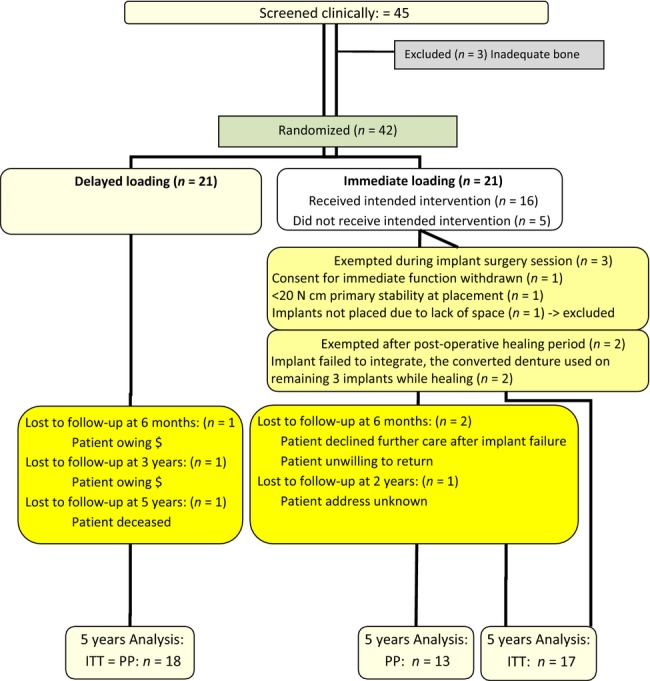
CONSORT Flow diagram of study participants through the trial.

Since the baseline, six participants have dropped out of the trial. In the control group, three participants dropped out at 6 months and 3 years due to unpaid bills and one participant died after the 4-year examination of causes unrelated to the dental implant. In the experimental group, one participant declined further treatment following an implant failure and another failed to come in for follow-up examinations upon completion of the treatment. A third participant moved away from Toronto after the 1-year examination. Hence, at the 5-year post-implant placement, the control and the experimental study arm groups consisted of 18 and 17 using an *intention-to-treat* (ITT) classification, alternatively 18 and 13 participants using a PP classification (Fig.6).

The participants that completed the follow-up examination 5 years after implant placement demonstrated relatively similar clinical and demographic characteristics as measured at the study baseline (Table2). No statistical differences between the study arms could be identified (*P* > 0.05).

**Table 2:** Baseline data of the participants that completed the 5-year follow-up examinations (*n* = 35/42 participants)

	Control group (ITT = PP; *n* = 18 participants, 72 implants)	Experimental group (PP; *n* = 13 participants, 52 implants)	Originally allocated to experimental group, but intended intervention not received (*n* = 4 participants, 16 implants)
Gender males, *n* (%)	9 (50)	9 (69)	2 (50)
Age at implant placement, years, Mean (SD) (min–max)	62 (9) (47–78)	62 (11) (42–82)	71 (3) (69–74)
Years edentulous, years, Mean (SD) (min–max)	8 (12) (1–43)	10 (13) (1–35)	1 (0.3) (0.5–1)
Smoking, Current or former, *n* (%)	11 (61)	6 (46)	3 (75)
Dental status in maxilla, Dentate–Full Denture–Partial Removable–Implant-retained prosth.	1-13-3-1	0-9-3-1	0-4-0-0
Bone quality (I–IV), *n*	18-36-18-0	0-16-36-0	8-8-0-0
Bone form knife (K)–parallel (P)–taper (T)–undercut (U), *n*	36-10-22-4	20-20-12-0	8-0-0-8
Implant diameter (3.3–3.75–4 mm), *n*	0-64-8	1-45-6	0-15-1
Implant length (10–11.5–13–15 mm), *n*	1-4-5-62	5-9-38	6-3-7

A participant in the control study arm group experienced that a previously osseointegrated implant at the 5-year examination in 2012 had become loose due to loss of bone. The participant had demonstrated very poor oral hygiene in spite of continuous attempts of remotivation. Moreover, the participant had been diagnosed with diabetes in 2010 and used multiple medications. Radiographic evidence showed a 7 mm bone loss occurring around the failing implant between 2010 and 2012.

The periapical radiographs taken immediately after surgery for implant location verification purposes were compared with the standardized periapical radiographs taken at the baseline. It could not be established that there were any significant differences in early bone loss, showing 0.5 mm (SD = 0.4) in both the experimental group and the control group, albeit it was recognized that the non-standardized nature of the radiographs made these comparisons problematic.

**Figure 7:**
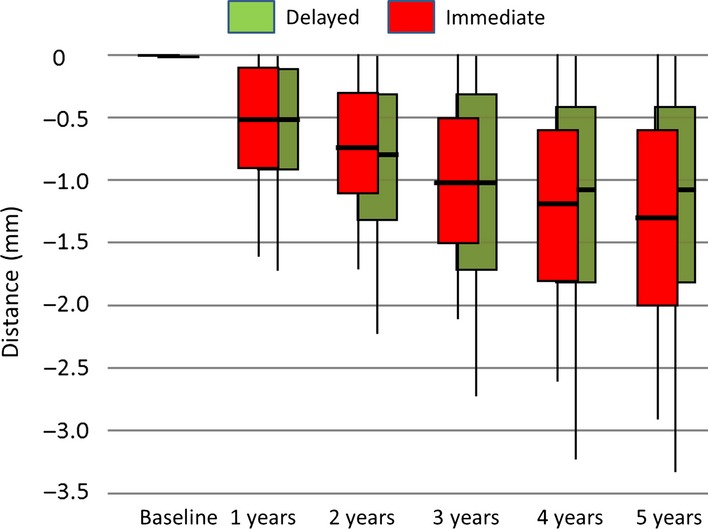
Crestal bone loss measured radiographically from baseline to 5 years following the implant placement (*P *> 0.05).

The bone level changes between baseline and 5 years were 1.2 mm (SD = 0.7) for all the implants, with no statistical differences between the experimental and the control study arms (Fig.7). The change from baseline was statistically significant in both study arms. The average bone loss over 5 years among the four implants supporting the full-arch FDP was 1.3 mm (SD = 0.7) for the medial pair of implants and 1.2 mm (SD = 0.7) for the two most distal implants. Thus, the distal cantilever did not seem to accelerate bone loss on the most distally placed implants (Table3).

**Table 3:** Radiographic bone loss measured at the 5-year follow-up examination (*n* = 35/42 participants, 140 implants, 280 mesial and distal measurement sites)

	Control group (ITT = PP; *n* = 18p/144i sites)	Experimental group (PP; *n* = 13p/104i sites)	Originally allocated to experimental group, but intended intervention not received (*n* = 4p/32i sites)
Bone loss, *n* (column %)			
<1 mm	57 (40)	33 (32)	12 (38)
1 to <2 mm	67 (49)	48 (46)	14 (44)
2 to <3 mm	15 (10)	23 (22)	6 (19)
>3 mm	3 + 2*	0	0
Mean bone loss (SD) (max–min)	1.1 (0.7) (0–3.3)	1.3 (0.7) (0–2.9)	1.2 (0.6) (0.3–2.6)

* One implant removed at the 5-year follow-up observation.

**Figure 8:**
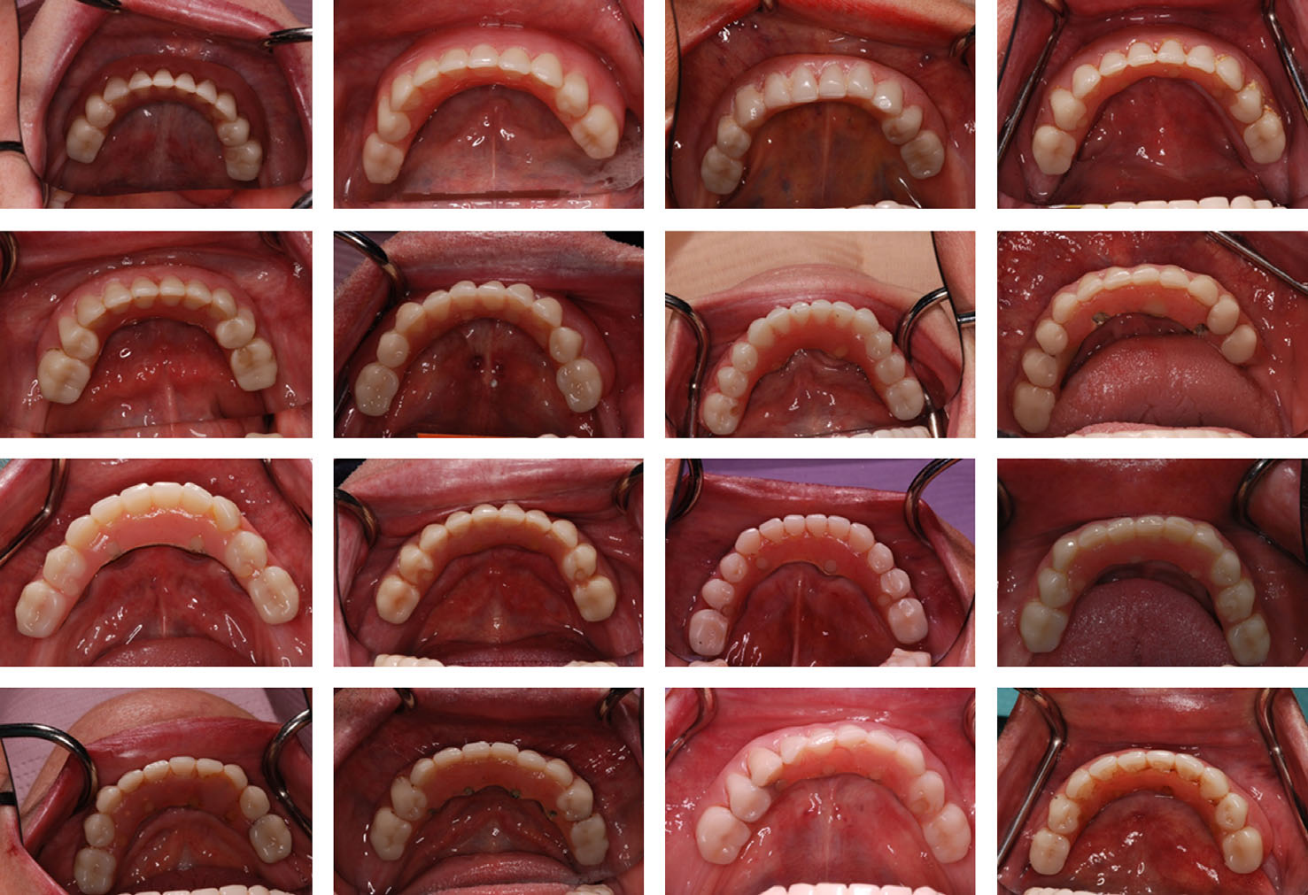
Representative fixed dental prostheses after 5 years.

Maintenance needs were fairly similar for both study arms as regards type of adverse events as well as incidence (Table4, Fig.8). The oral hygiene and the occurrence of sulcus bleeding did not differ significantly between the two study arm groups.

**Table 4:** Participants having experienced post-operative biological and technical adverse events following the implant placement by time to event (pre-loading period–Loading–>Year 2–Year 2+)

	Control group (ITT = PP; *n* = 18)	Experimental group (PP; *n* = 13)	Originally allocated to experimental group, but intended intervention not received (*n* = 4)
No adverse events whatsoever during first 5 years	7	2	0
Soft-tissue adverse events			
Transient pain or Sensory disturbance	0–1–2	0–0–1	0–0–0
Transient swelling	0–1–1	0–0–0	0–1–1
Bleeding on probing	0–0–7*	0–0–4	0–0–2
Implant system adverse events			
Loosening of implant	0–0–1†	0–0–0	2–0–0
FDP adverse events			
Surface fractures (Most commonly tooth fractures)	1–3–3‡	3–2–4§	0–0–2

Sums exceed sample size because some participants experienced multiple different problems.

* One participant accounted for seven soft-tissue adverse events.

† Implant removed in 2012.

‡ One participant accounted for eight fixed dental prosthesis (FDP) adverse events.

§ One participant accounted for six FDP adverse events.

## Discussion

One satisfying aspect of this trial is that 35 of the original 42 participants remained in the study over the 5 years. The statistical analyses were primarily focused on the PP data and not in the ITT data, which is a decision open for debate. Moreover, four participants in this trial were exempted from the experimental arm, but they were not considered as reallocated to the control group, nor excluded. In a correct ITT analysis, everyone who begins an intended treatment is considered to be part of the trial, whether he or she finishes it or not. Hypothetically, in this trial, the participants exempted from the experimental group could on one hand be defined as “failures,” which is technically correct if defined by a criterion that the participant should have received four implants and a fully functional FDP retained by these four implants immediately following the intervention. On the other hand, one may argue that having received four implants that heals properly, although not necessarily together with an immediately functional FDP retained by all four implants, is also a satisfactory outcome, particularly if regarded from a patient lifelong perspective. A problem with participant reassignments in implant studies is that participants in the experimental group may not actually receive treatment at all. On the other hand, there are weaknesses when participants not eligible for treatment are included in the outcome of the treatment. For example, a necessary minimum primary stability was never reached or some form of complication during surgery occurred. One solution may be perhaps to exclude all those not eligible for immediate function from the study as a study inclusion criterion and randomize after implant stability has been assessed. However, this would exclude from both groups and reduce the study power and the relevance to clinical practice can also be questioned.

The inclusion and exclusion criteria were considered relatively broad and comparable to other clinical studies. Some studies require participants with specific widths of keratinized mucosa, which was ignored in this trial. Albeit neither type four bone, or skeletal discrepancies were used as exclusion criteria, neither characteristic was observed among the current participants.

The <10 cigarettes per day smoking requirement was perhaps on the stricter side, compared with other authors that have allowed much more. The randomization process appeared to generate fairly equal study arms apart from a tendency toward imbalance in gender and smoke status (Table2). These imbalances are probably coincidental and considered inconsequential.

In the current trial 3, implants failed to osseointegrate in the experimental study arm, compared with one implant in the control arm, which may indicate that immediate loading may be associated with a slightly higher risk of unsuccessful osseointegration. This observation corroborates the conclusions in a systematic review of clinical studies that reported on the implant survival and success of immediate versus delayed loading suggesting that there is a slight tendency to favor the delay loading with a pooled difference of 2% (CI 0–4%) better survival using the latter approach (Jokstad & Carr 2007). However, this estimate was based mainly on the clinical studies having compared traditional machined-surfaced titanium implants, and it was recognized that the conclusion may not be representative for modern implant surfaces. Moreover, the effect of the relatively low minimal insertion torque used in the current study could have had an influence on the results. In the current trial, a minimum of 20 N cm was selected, based on clinical studies at the time of study initiation (Glauser et al. 2003, 2005; Rocci et al. 2003; Attard et al. 2005; Jungner et al. 2005; Watzak et al. 2006), while the manufacturer in their 2005 product catalog recommended immediate loading for the particular implant used in this trial within the range of 35–45 N cm (Nobel Biocare 2012). That single implants meant to be immediately loaded requires a minimum initial stability, represented by a minimum insertion torque value or ISQ as a function of implant design sounds reasonable, and has been demonstrated clinically (Ottoni et al. 2005). However, the biological rationale for a particular minimum insertion torque of implants rigidly splinted by an immediate supraconstruction remains to be clarified (Javed & Romanos 2010; Javed et al. 2011).

The most common adverse events were surface fractures of the FDPs as well as bleeding on probing. These are common problems associated with the particular FDP design used in this trial (Bryant et al. 2007). In this regard, it is imperative to design the FDPs with confluent convex surfaces and particularly the intaglio surface to allow the participant to maintain adequate oral hygiene. However, although the participants were repeatedly remotivated and instructed for good oral hygiene at each follow-up and supplied with interdental brushes, the calculus buildup in the mandible formed readily in several participants and had to be removed mechanically each year. Remarkably, these participants experienced little or no bone loss, which underscores that the amount of plaque is on its own not directly proportional to accelerated bone loss.

In the current study, we measured a bone loss of 0.5 mm in both study arms in the period between the implant placement and the baseline radiographs. However, the bias introduced due to comparing non-standardized radiographs is acknowledged. In several studies, the comparison of crestal bone levels between immediate and delayed loading is confounded by the fact that the baseline radiographs are taken immediately following the implant placement and first after 3 or 4 months, respectively (Jokstad et al. 2011). There is limited information about how bone loss progresses in this early phase following implant placement, but it has been proposed that it is more pronounced during the first and second months with estimates of about 0.4–0.6 mm (Brägger et al. 1992; Brägger 1998) and even more if the implant is placed deeper into the bone (Hämmerle et al. 1996).

The fit of the conversion prosthesis to the implants was verified clinically at the time of delivery on the same day as the implant surgery, but not measured numerically. Theoretically, the polymerization shrinkage of the acrylic material used to lute the temporary abutments to the pre-existing denture could introduce an imprecise fit. A few years after the study had commenced the temporary FDPs that had been collected and archived by the investigators were matched to the implant replicas in the dental stone casts made from the impressions taken for the fabrication of the final FDPs. No discrepancies or misfits were observed. Although this does not necessarily exclude the possibility of imprecision on account of incorrect dental stone cast, the fact that an intraoral acrylic jig had been used to verify the implant replica positions in dental stone cast lend credence to the assumption that the fit of the temporary FDPs converted from the pre-existing denture were indeed acceptable. Another consideration is that even if there was a misfit, one would expect that some degree of flexure of the acrylic would be expected due to the low elasticity of modulus of the material. Under such conditions, one would expect breakage sooner or later, depending on degree of misfit, denture thickness, occlusion and chewing forces. Regardless of the exact degree of fit between the conversion FDPs and the implants, the current trial data suggest that using a converted denture for 3 months is not associated with multiple mechanical or technical problems. An exception is the “cluster failure” participant, of which three were identified in the current trial experiencing 8, 7 and 6 events, respectively, involving some form of fractures of both the temporary and permanent FDPs (Table4).

The innovative idea of converting a pre-existing full denture to a temporary fixed bridge can be attributed to Thomas J. Balshi, who originally launched the concept as the “Biotes conversion prosthesis” (Balshi 1985). The prosthesis was meant to be used with an addition of periodontal dressing around the implants following the stage 2 surgery. Subsequent refinements made by the investigator involved replacing the original gold cylinders with modified square impression copings (Balshi & Wolfinger 1996), converting the denture following the stage 1 instead of the stage 2 surgery (Balshi & Wolfinger 2002) and finally refining the solution into their trademarked “Teeth in day” method (Balshi & Wolfinger 2003).

Another immediate function approach introduced around this period by Maló et al. (2003) was the “All-on-Four” treatment concept for the edentulous mandible. A difference between the current trial and the all-on-four concept is the idea in the latter to tilt the two distal implants 45° distally combined with angulated abutments to reduce the length of the cantilever and/or allow 12 units in the FDP. The original 2003 protocol involved an impression and a rapid fabrication of an acrylic resin FDP within 2 h, although later refinements involve both a prefabricated adaptable FDP as well as all-on-six approaches in the maxilla (Pomares 2009). There are benefits and disadvantages in terms of costs, logistics and complexity of using the approach detailed in the current study versus an all-on-four solution.

Perhaps the strongest argument for proposing the use of the conversion prosthesis, approach is the current developments within the field of digital prosthodontics. A 3D scan of the post-converted denture, and even the pre-converted denture in case of anticipated risks of a dubious healing event, can be performed in any modern desktop scanner either after the completion of the initial intraoral fitting or alternatively following one or more adjustments during the course of the healing period and when it is the most convenient for the participant. Once the record has been made, any additive or subtractive fabrication method can be used to print or mill from the STL file, an exact replica in whatever biomaterials are preferable.

## Conclusion

Same day loading of implants in the anterior mandible to retain a full-arch FDP converted from a pre-existing optimal denture compared with waiting for 3–4 months before loading seems to yield comparable outcomes after 5-year observation.

## Supporting Information

**Data S1.** CONSORT Statement 2001 Checklist (http://www.consort-statement.org/mod_product/uploads/CONSORT2001checklist.pdf).
